# Comparison of the fecal microbiota of domestic commercial meat, laboratory, companion, and shelter rabbits (*Oryctolagus cuniculi*)

**DOI:** 10.1186/s12917-018-1464-6

**Published:** 2018-04-27

**Authors:** Jennifer Kylie, J. Scott Weese, Patricia V. Turner

**Affiliations:** Department of Pathobiology, University of Guelph, Guelph, ON N1G 2W1 Canada

**Keywords:** Gastrointestinal microbiota, Rabbit, Rabbit enteritis complex

## Abstract

**Background:**

Rabbits are cecotrophic, hindgut-fermenters that rely heavily on their gastrointestinal microbiota for optimal digestion of plant-based diets. Dysbiosis, caused by disruption of the gastrointestinal microbiota, is known to predispose rabbits to rabbit enteritis complex (REC), a major cause of morbidity and mortality. The objectives of this study were to describe the fecal microbiota of domestic rabbits from a variety of settings (commercial meat, companion, laboratory, and shelter) and to identify how factors such as age, season, and routine antimicrobial use affect the fecal microbiota composition.

**Results:**

A total of 86 pooled commercial meat, 54 companion, 14 pooled laboratory, and 14 shelter rabbit fecal samples were evaluated using 16S rRNA gene sequencing of the V4 region. In all sample types, the predominant bacterial phylum was Firmicutes. Other commonly identified phyla (composing ≥ 1% of the total microbiota composition) were Verrucomicrobia, Proteobacteria, and Bacteroidetes. Significant differences in composition were noted between commercial, companion, laboratory, and shelter rabbit samples for proportions of Verrucomicrobia (*P* < 0.01), Proteobacteria (*P* < 0.01), and Lentisphaerae (*P* = 0.01) within the total microbiota. Within the commercial meat rabbit samples, significant differences between the microbiota composition of growers (*n* = 42) and does (*n* = 44) were limited to one unclassified Firmicutes (*P* = 0.03) and no differences were identified at the phylum level. Significant differences were present between fecal samples taken from rabbits during the summer (*n* = 44) compared to the winter (*n* = 42), with Firmicutes (*P* = 0.04), Verrucomicrobia (*P* = 0.03), Proteobacteria (*P* = 0.02), Deinococcus-Thermus (P = 0.04), Armatimonadates (*P* = 0.003), and Actinobacteria (P = 0.03) forming significantly different proportions of the microbiota. The only significant difference in composition between those farms that routinely reported antimicrobial use and those that did not was in one unclassified Bacteroidetes (*P* < 0.05) and no differences were identified at the phylum level.

**Conclusions:**

Rabbit husbandry and diet, in addition to season, significantly influence the fecal microbiota composition of domestic rabbits, while age of the rabbit post-weaning has minimal impact.

**Electronic supplementary material:**

The online version of this article (10.1186/s12917-018-1464-6) contains supplementary material, which is available to authorized users.

## Background

Rabbits are herbivorous, monogastric, hindgut-fermenting mammals that rely on cecotrophy to ensure maximum nutrient absorption from their diet. Management of this unique gastrointestinal (GI) physiology can be challenging, and, as a result, enteric disease is common in domestic rabbits. One of the most difficult aspects of managing rabbit digestion is maintaining the normal commensal gastrointestinal microbiota. In rabbits, disruption of the normal microbiota, termed dysbiosis, is commonly implicated as a cause of enteritis, with ensuing diarrhea, subsequent dehydration, inadequate nutrition, and, potentially, death as a result.

The etiopathogenesis of enteritis in rabbits is complex and generally multifactorial. Enteric pathogens such as *Escherichia coli*, *Clostridium spiriforme*, *Lawsonia intracellularis*, rotavirus, and coronavirus are commonly associated with diarrhea outbreaks in rabbits, as are other agents such as *Clostridium piliforme*, *Salmonella* spp*.*, parvovirus, and astrovirus [1–6]. However, much is still unknown about initiating factors as infection with these organisms is not synonymous with disease and subclinical infection may occur with no overt clinical signs [7]. Most cases of enteritis in rabbits are caused by a combination of factors, including feeding of a low fiber diet, debility and overall health status, management-related stress, and age, along with the presence of one or more potentially pathogenic organisms [7–9].

Because of its critical importance in rabbit health, several studies have focused on understanding the composition of the rabbit enteric microbiota. Historically, researchers used culture-based techniques; however, the development of culture-independent techniques has permitted much greater in-depth analysis [10, 11]. Culture-based studies concluded that Bacteroidetes was the predominant bacterial phylum within the rabbit gastrointestinal tract, regardless of age [12]. With culture-independent sequencing Bacteroidetes are still reported as predominant in the intestinal tract of very young rabbits, but the predominant phylum in post-weaned and adult rabbits is Firmicutes, with Bacteroidetes accounting for only a small fraction [13–16]. Other predominant phyla routinely identified using culture-independent sequencing include Verrucomicrobia and Proteobacteria [14, 16].

Previous studies examining the fecal microbiota of rabbits have used small numbers of laboratory rabbits kept in well-controlled environments exposed to minimal environmental or husbandry variations, making broad applicability to other domestic rabbit conditions less likely. Factors such as diet, husbandry, and seasonal effects have been demonstrated to have effects on the fecal microbiota in both humans and other animal species [17–19], but have not been explored in rabbits. The objectives of this study were to characterize the microbiota of domestic rabbits kept in a variety of environments and to identify factors contributing to enteric microbiota variations using a culture-independent, high-throughput sequencing method. We hypothesized that significant fecal microbiota differences would be seen between commercial meat rabbits and domestic rabbits kept in other settings, largely because of differences in husbandry, including dietary composition, routine use of antimicrobials, and environmental management. Additionally, we hypothesized that these differences would be least significant between commercial meat rabbits and rabbits housed in a shelter environment (i.e., a facility where previously-owned animals have been surrendered for re-homing) due to the likely inconsistencies in the environment of both of these types of rabbits and diet (in the case of shelter rabbits), while differences would be most significant between commercial meat rabbits and both companion and laboratory rabbits due to more consistent environmental conditions, diet, care, and reduced infectious disease within these latter two groups of rabbits.

## Results

A total of 168 rabbit fecal samples from various sources were included in the final analysis: 86 pooled commercial meat, 54 companion, 14 pooled laboratory, and 14 shelter animals. Twenty-three samples (14 commercial meat, 8 companion, and 1 shelter) were not included in the final analysis either due to an inability to produce bands following DNA extraction, amplification, and purification, an insufficient quantity of DNA within the sample (< 12.5 nM) at the time of normalization prior to sequencing or an insufficient number of sequences (< 5000) present following the completion of all quality control filters. A total of 10,897,154 V4 16S rRNA gene sequences passed all quality control filters. Sequence numbers per sample ranged from 6359 to 325,029, with a mean of 64,864, median of 53,721, and a standard deviation of 46,279.

### The domestic rabbit fecal microbiota

The analysis was based on 168 samples, with a subsample of 4577 sequences per sample for normalization. A total of 31 bacterial phyla were identified; however, only five were present at a relative abundance of ≥ 1%. Of these five phyla, Firmicutes composed 66.4% of the total microbiota, Verrucomicrobia composed 14.1%, Proteobacteria composed 9.5%, and Bacteroidetes composed 1.54% while 6.9% of the sequences could not be identified at the phylum level (see Additional file 1: Table S1 for further information).

### Comparison of the rabbit fecal microbiota based on animal source

**Fig. 1:**
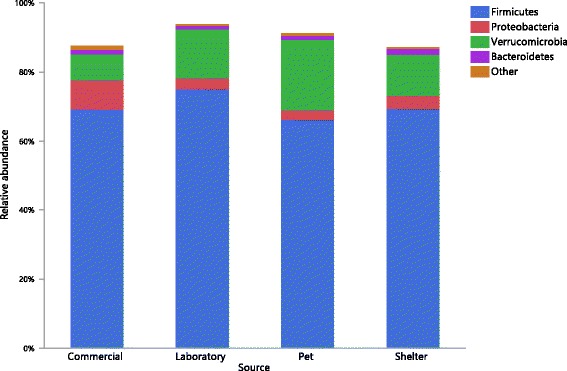
Median relative abundances of predominant bacterial phyla of the rabbit fecal microbiota separated by animal source. Significant differences (*p* ≤ 0.05) are present between sources for Proteobacteria, Verrucomicrobia, and Lentisphaera (included in ‘Other’ and composed < 1% of the total composition)

There were several statistically significant differences in relative abundance of bacteria within all taxonomic levels based on animal source. These differences remained consistent regardless of whether the specific source from which the sample came was included as a random effect in the analysis; therefore, all relative abundance analyses that are reported below exclude specific source as a random effect. Significant differences were identified between commercial rabbits, and companion and laboratory rabbits in relative abundances of Proteobacteria (*P* < 0.01), between commercial rabbits, companion rabbits, and laboratory and shelter rabbits in relative abundances of Verrucomicrobia (*P* < 0.01), and between commercial rabbits, and companion and shelter rabbits in relative abundances of Lentisphaera (*P* < 0.01) when the *p*-value was corrected for false discovery rates (Fig. 1). These differences were also reflected at all levels of taxonomic classification (see Additional file 2: Table S2).

**Fig. 2:**
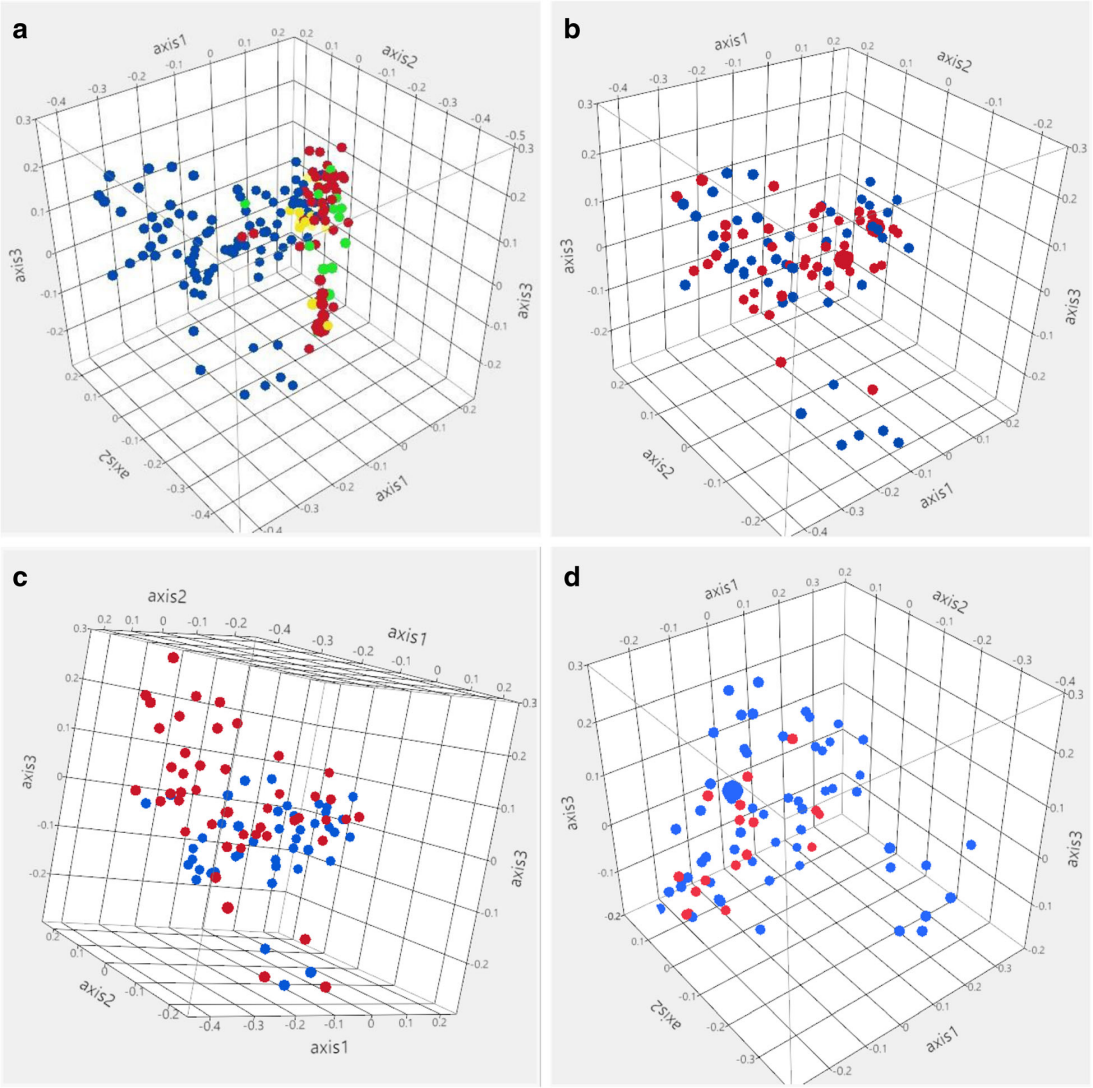
Principle co-ordinates analysis (PCoA) of the rabbit fecal microbiota based on the Jaccard Index. **a** blue circles = commercial meat rabbits, red circles = companion rabbits, yellow circles = laboratory rabbits, green circles = shelter rabbits; **b**) blue circles = does, red circles = fryers; **c**) blue circles = winter, red circles = summer; **d**) blue circles = routine antimicrobial use, red circles = no routine use of antimicrobials. Clustering of markers within a plot indicates similarity in microbiota community membership. Significant differences are present between rabbit sources and between seasons, but not between ages and antimicrobial use statuses

**Fig. 3:**
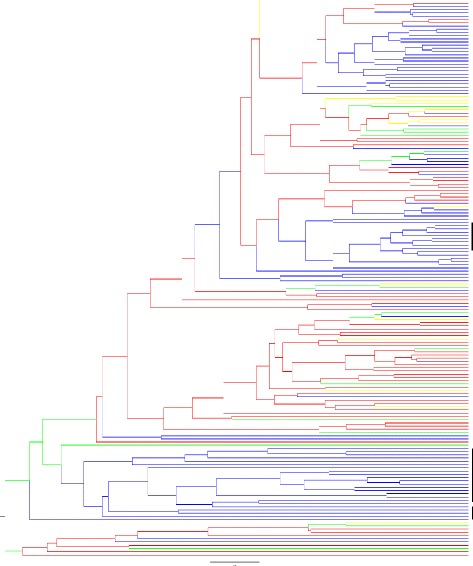
Dendrogram of the rabbit fecal microbiota community structure based on the Yue and Clayton Index of Dissimilarity. Blue = commercial rabbits, red = companion rabbits, yellow = laboratory rabbits, green = shelter rabbits. Clustering of lines indicates similarity in microbiota community structure. Significant differences are present between commercial meat rabbits and all other sources. The top cluster delineated by the vertical black line indicates clustering of samples collected from commercial meat rabbits during the winter months while the clusters indicated by the lower vertical black lines indicate clustering of samples collected from commercial meat rabbits during the summer months

Commercial meat and companion rabbits had distinctly different fecal microbiota community structures, while those of laboratory and shelter rabbits tended to overlap with those from companion rabbits (Fig. 2). The dendrogram of community structure (Yue and Clayton index of dissimilarity) is presented in Fig. 3.

In both cases, clustering of ≥ 10 samples can be seen within large numbers of commercial and pet samples, with the laboratory and shelter rabbit fecal samples scattered intermittently between, and primarily amongst, the companion samples. Using the Jaccard tree, significant differences were noted between all possible group pairings using a parsimony test (*P* < 0.02 for all comparisons), something that was also noted with unweighted UniFrac tests (*P* ≤ 0.04 for all comparisons), indicating that the fecal microbiota community membership differed significantly between all sources. Using the parsimony test with the Yue and Clayton tree, significant differences were identified between commercial meat and laboratory samples (*P* = 0.03), commercial meat and companion animal samples (*P* < 0.01), and commercial meat and shelter samples (*P* < 0.01). These differences were also detected using unweighted UniFrac (all *P* < 0.01), indicating that fecal microbiota community structure was significantly different between commercial meat rabbits and rabbits from all other sources, but the other sources did not differ amongst one another. Thirty-seven OTUs were identified to be differentially abundant via LEfSe (*P* ≤ 0.05) with LDA scores ≥ 3.0 (Table 1).

**Table 1:** OTUs identified as significantly differentially abundant (*p* ≤ 0.05) from all other rabbit sources with LDA scores ≥ 3.0

Commercial meat	Companion	Laboratory	Shelter
Unclassified Ruminococcaceae *Tissierella* Unclassified Aerococcaceae *Erysipelothrix* *Facklamia* Unclassified Pseudomonadaceae *Paenalcaligenes* *Ignatzschineria* *Oligella* *Escherichia_Shigella* *Oceanisphaera* *Stenotrophomonas*	Unclassified Bacteroidetes Unclassified Lachnospiraceae Unclassified Bacillaceae_2 *Campylobacter* Unclassified Desulfovibrionaceae *TM7_genus_incertae_sedis* *Persicirhabdus* Unclassified Verrucomicrobiaceae	Unclassified Firmicutes Unclassified Clostridia *Flavonifractor* *Pseudoflavonifractor* *Anaerostipes* *Lutispora* *Lachnospira* *Acetivibrio* Unclassified Alphaproteobacteria Unclassified Betaproteobacteria	*Corynebacterium* Unclassified Bacteroides *Barnesiella* *Acetitomaculum* Unclassified Erysipelotrichaceae *Bacillus* *Pseudomonas*

### Comparison of the rabbit fecal microbiota based on animal age

Forty-four samples, each consistently of pooled feces from 3 does per commercial meat farm, and 42 samples composed of pooled feces from 3 growers per commercial meat farm, were compared, with a subsample of 4577 sequences per sample. No significant differences were observed in relative abundance between the two age groups at the bacterial phylum, order, family, and genus levels (all adjusted *P* > 0.05). At the class level, there was a significant difference between does and growers for an unclassified Firmicutes, where the relative abundance in does was 5.8% and in growers was 7.7% (*P* = 0.03).

**Fig. 4:**
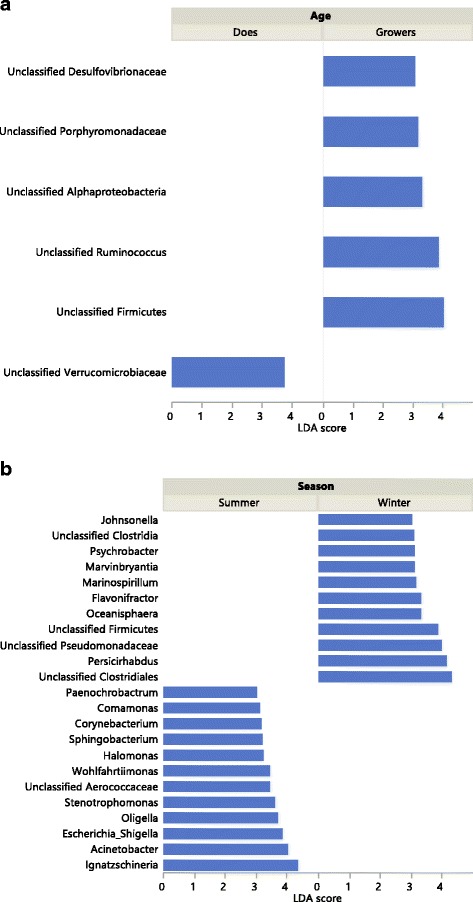
OTUs noted to be significantly different between **a**) ages and **b**) seasons with LEfSe scores > 3.0

Community structure differences are visualised in the PCoA (Fig. 2b). There is significant overlap between the community structures of the two different age groups with no obvious grouping based on age. A significant difference between the fecal microbiota community structure of does and fryers was only noted using the Yue and Clayton tree with the parsimony test (*P* = 0.04); however, no more than five samples were noted to be clustered by age within commercial meat samples in the Yue and Clayton tree analysis (Fig. 3). Six OTUs were identified to be significantly different between ages (*P* ≤ 0.05) with LEfSe LDA scores ≥ 3.0 (Fig. 4a).

### Comparison of the rabbit fecal microbiota based on season

A comparison was conducted of 44 pooled fecal samples collected from meat rabbits during winter months and 42 pooled fecal samples collected during summer months, with a subsample of 4577 sequences per sample. Significant differences at all taxonomic levels of classification were identified (Table 2).

**Table 2:** Relative abundance and FDR *p*-values for significantly different (*P* ≤ 0.05) samples from summer (*n* = 44) and winter (*n* = 42)

	Relative Abundance - Summer	Relative Abundance – Winter	FDR *P*-value
Phylum			
Actinobacteria	1.14%	0.57%	0.03
Bacteroidetes	1.90%	1.22%	0.03
Deinococcus-Thermus	0.06%	0.01%	0.04
Proteobacteria	18.57%	9.09%	0.02
Verrucomicrobia	7.29%	10.64%	0.03
Firmicutes	62.94%	69.51%	0.04
Order			
Actinomycetales	0.82%	0.17%	0.04
Flavobacteriales	0.33%	0.09%	0.04
Rhodobacterales	0.20%	0.04%	0.04
Xanthomonadales	5.86%	0.95%	0.04
Family			
Microbacteriaceae	0.06%	0.01%	0.03
Flavobacteriaceae	0.30%	0.06%	0.03
Rhodobacteraceae	0.20%	0.04%	0.05
Burkholderiaceae	0.01%	< 0.01%	0.01
Moraxellaceae	2.20%	0.55%	0.03
Xanthomonadaceae	5.86%	0.95%	0.03
Aerococcaceae	1.19%	0.38%	0.03
Unclassified Clostridiales (Firmicutes)	17.7%	22.2%	0.05
Genus			
*Leucobacter*	0.04%	0.01%	0.05
*Flavobacterium*	0.07%	< 0.01%	0.03
*Myroides*	0.11%	0.01%	0.05
*Burkholderia*	0.01%	< 0.01%	0.02
*Delftia*	< 0.01%	< 0.01%	0.03
Unclassified Enterobacteriaceae (Proteobacteria)	0.11%	0.01%	0.05
*Acinetobacter*	2.16%	0.38%	0.02
*Serpens*	0.04%	< 0.01%	0.04
*Wohlfahrtiimonas*	0.41%	0.02%	0.05
*Marvinbryantia*	0.19%	0.34%	0.02

The community structure differences are presented in the PCoA in Fig. 2c. While there is significant visible overlap between the groups, there is a distinct subpopulation of samples from the summer group that is separate from the overlying samples. Significant differences were identified between summer and winter fecal microbiota community membership and structure using a parsimony test (*P* < 0.01), and unweighted UniFrac tests (*P* < 0.01) with both the Jaccard tree and the Yue and Clayton tree (*P* < 0.01 for both tests). One distinct cluster comprised of ≥ 10 commercial meat rabbits samples collected during the winter months was present, as was one distinct cluster of ≥ 10 samples collected during the summer months within the Yue and Clayton Tree calculated based on animal source (Fig. 3). Twenty-three OTUs were identified to be significantly different between season (*P* ≤ 0.05) with LDA scores ≥ 3.0 (Fig. 4b).

### Comparison of the rabbit fecal microbiota based on routine antimicrobial use

Routine use of antimicrobials in feed or water was reported in 17 of 23 (73.9%) farms, while 3 farms did not respond and one farm had closed at the time of follow-up questioning. Farms for which antimicrobial use could not be confirmed were excluded from the analysis. Antimicrobials reported to be used routinely included, in order of descending frequency of use, salinomycin sodium 6%, virginiamycin, tylosin, chlortetracycline, bacitracine methylene disalicylate, sulfamethazine, sulfadimethoxine, and one anti-coccidial agent that was not further specified. Twelve of the 17 (70.6%) farms reporting routine antimicrobial use reported using ≥ 2 antimicrobials in combination, making statistical analysis of the data beyond the general category of “routine antimicrobial use” not feasible. Antimicrobials were not reported to be routinely used in any of the laboratory rabbits sampled, and antimicrobial use could not be confirmed for the companion or shelter rabbit samples; thus, results from these groups were also excluded from this analysis.

Seventy-five pooled commercial meat rabbit fecal samples were compared on the basis of routine antimicrobial use following sequence filtering and the removal of non-responders, 58/75 (77.3%) with routine antimicrobial use, with a subsample of 4577 sequences per sample. The only significant difference observed at all levels of taxonomic classification, aside from the phylum level where no significant differences were observed, was in one Bacteroidetes unclassified at the class level (*P* < 0.01 at all taxonomic levels), which was significantly higher in samples from farms where no routine antimicrobial use occurred (0.85, 0.75, 0.75, 0.57% at class, order, family, and genus levels, respectively) compared to when routine use did occur (0.29, 0.31, 0.30, 0.32%, respectively).

No significant differences in community membership or structure were identified using Jaccard or Yue and Clayton trees with both the parsimony and unweighted UniFrac tests (*p* ≥ 0.29 for all tests); samples from farms that did not report routine use of antimicrobials can be seen as well-distributed amongst samples from farms that did in the PCoA in Fig. 2d. Thirteen OTUs were identified to be significantly different between use status (*P* ≤ 0.05) with LDA scores ≥ 2.0; single OTUs with an LDA score ≥ 3.0 that were more frequently identified with routine antimicrobial use were from each of the following genera: *Ignatzschineria, Erysipelothrix*, and *Wohlfahrtiimonas*. One OTU with an LDA score ≥ 3 was significantly more prevalent when antimicrobials were not being routinely used was present from each of the following genera: *Acetitomaculum, Clostridium* cluster III, and *Parasporobacterium*, in addition to one unclassified Ruminococcaceae, one unclassified Desulfovibrionaceae, and one unclassified Bacteroidetes.

## Discussion

Our study provides significant insight into the composition of fecal microbiota of domestic rabbits, as well as factors that may promote changes in its composition. The large number of animals sampled, combined with the variety of factors incorporated into the study, suggests that the results are applicable to rabbits in a variety of domestic settings. Consistent with previous culture-independent sequencing studies, such as those conducted by Eshar and Weese [14] and Zhu et al. [16], the predominant phylum identified in the rabbit fecal microbiota, regardless of source, age, season or reported antimicrobial use was Firmicutes. Within this phylum, the Clostridia were the most abundant class identified, of which the Ruminococcaceae and the Lachnospiraceae were the most abundant families present. While the prevalence of Firmicutes in our study is lower than that reported by Eshar and Weese (66% vs. 82%, respectively), other studies report levels of Firmicutes varying between 61 and 82% with significant individual variation, suggesting that the relative abundance of Firmicutes in the rabbit enteric microbiota falls somewhere within this range [16, 20]. In addition to being identified as the predominant phylum present in the rabbit gut, Firmicutes has been described as the most abundant phylum in the gastrointestinal microbiota of most healthy adult mammals, including humans [21–24], emphasizing its likely importance as a commensal in GI health.

Other predominant phyla identified include Verrucomicrobia, Proteobacteria, and Bacteroidetes. The relative levels identified for these phyla are consistent with previous reports, although proportions of Verrucomicrobia appear to vary considerably with the method of analysis [14, 16, 20, 25]. While Verrucomicrobia is still a relatively newly-defined group of organisms, *Akkermansia* (within this phylum) has been suggested to have a key role in hydrolysis of diverse ingested polysaccharides, contributing to more complete digestion of dietary cellulose as well as methane metabolism [26–28]. Thus, its presence as an important constituent of the rabbit fecal microbiota is unsurprising. The most well-known species in this genus is *Akkermansia muciniphila*, a mucin-degrading bacterium that has been demonstrated to be beneficial in both human and animal health [29, 30]. For example, increases in the proportion of fecal *Akkermansia mucinophila* have been associated with a healthier metabolic status in both humans and mice [29, 30]. While the impact of *Akkermansia muciniphila* on rabbit health is still unknown, it is known that the ability to breakdown mucin is especially important during cecotrophy for optimal nutrient extraction [8]. In vitro studies have also identified a positive correlation between the proportion of Verrucomicrobiaceae and concentrations of proprionate and acetate, volatile fatty acids that are found in high concentrations within rabbit ceca following the breakdown of fiber-rich carbohydrates, and significant energy sources [20, 31]. *Akkermansia mucinophila* levels have also been inversely correlated with several inflammatory markers in mice [32], indicating that a decrease in *Akkermansia mucinophila* levels may result in a more pro-inflammatory gut environment. A decreased proportion of Verrucomicrobia within the fecal microbiota of commercial meat rabbits overall, and particularly during the summer months, as found in this study, suggests less optimal gut health and nutrient extraction in this group of rabbits, as well as a pro-inflammatory state. This most likely reflects the lack of hay in the diet of commercial meat rabbits, compared to rabbits from other sources, as well as metabolic distress potentially due to heat stress, discussed in further detail below.

While small proportions of Proteobacteria are routinely observed within the enteric microbiota of all mammals, it has been suggested that increased relative abundance of Proteobacteria should be considered as a diagnostic indicator of underlying dysbiosis, predisposing individuals to enteric disease or indicating the presence of disease [33]. Relative increases in Proteobacteria with or without a concurrent reduction in Firmicutes have been observed in several species in cases of metabolic gastrointestinal disorders, such as genetically-related and diet-induced obesity [34, 35], as well as in chronic intestinal inflammation, such as inflammatory bowel disease and ulcerative colitis [36, 37]. It is hypothesized that the resultant bacterial population shift caused by increasing relative proportions of Proteobacteria stimulates the host gastrointestinal immune system, resulting in a pro-inflammatory response [38]. Therefore, the increased relative proportion of Proteobacteria observed within commercial meat rabbits overall, and specifically during the summer, suggests a shift away from the normal gut microbiota in these animals, increasing their risk for disease development. This idea is supported by the higher proportion of potentially pathogenic bacteria associated with REC identified in commercial meat rabbit fecal samples overall as well as in the summer at the OTU level. In both cases, elevations in *Escherichia/Shigella* accounted for significant differences in fecal microbiota composition when compared to feces from rabbits from other sources.

No significant differences were observed between does and growers in this study. This is consistent with previous reports suggesting that the rabbit fecal microbial community rapidly reaches a steady state at, or just after, weaning [20]. This stabilization has been observed in several other species, including mice [39] and pigs [40]. Additionally, no significant differences were observed between samples from farms in which antimicrobials were routinely used compared to farms where they were not. In 2007, Abecia et al. examined the effects of bacitracin and tiamulin on the cecal microbiota of lactating rabbit does. The study demonstrated that significant microbiota compositional changes were highly dependent on the specific antimicrobial used, with bacitracin having minimal effects on the cecal microbiota composition and tiamulin causing significant changes [41]. In the current study, a variety of antimicrobials with different pharmacologic mechanisms were reported in use alone or in combination, including those with anticoccidial activity and those used to treat respiratory disease. Effects of specific agents on the gut microbiota may have been masked by the concurrent use of other antimicrobial agents; additional studies into the effects of specific antimicrobial agents on the microbiota composition will help to differentiate those antimicrobials likely to have a significant effect versus those that have minimal effect.

Several factors that may contribute to gut microbiota composition are thought to differ significantly between domestic rabbit sources – in particular, the diet. Once animals are weaned, commercial meat rabbits are almost exclusively fed extruded pellet diets relatively low in fiber (15–20%) and high in carbohydrates to encourage rapid growth prior to slaughter [42]. This contrasts with recommended feeding practices for companion rabbits, which suggest a high fiber hay-based diet (e.g., timothy hay) that is supplemented (< 5%) by lower fiber pellets to ensure that all nutritional requirements are met while optimizing gastrointestinal function [9, 43]. Companion rabbit owners also frequently supplement this recommended dietary regimen with other fiber-rich fruits and vegetables. While the diet of laboratory rabbits is also primarily that of high carbohydrate pellets, the fiber content of many of the commercially available laboratory diets tends to be closer to 25%. Laboratory rabbits are also frequently given hay and vegetables as enrichment, although generally at more restricted levels than companion rabbits, while the dietary history of domestic rabbits in shelters is largely unknown. There is also significant variation in how long these animals may have been in a shelter receiving a balanced diet. Dietary fiber source, content, and digestibility are critical components of the rabbit diet, in which changes are directly and significantly correlated with enteritis and mortality in growing meat rabbits [44, 45]. It was beyond the scope of this study to evaluate specific changes in dietary fiber source, content, and type in relation to the rabbit fecal microbiota; however, this likely contributes to the majority of differences noted in microbiota between commercial meat and other sources of rabbits and is an area of further investigation.

Studies in other species examining the influence of season on the gut microbiota have identified decreases in the relative abundance of Verrucomicrobia during the summer as observed in the current study, but not relative increases in Bacteroidetes and Proteobacteria as were seen in this study [19]. The seasonal changes in the microbiota of Arctic ground squirrels observed in the Stevenson et al. 2014 study were attributed to diet availability and variation between seasons. In the current study, seasonality was only examined in commercial meat rabbits, in which the diet remains relatively consistent with fixed content proportions year-round (although changes in plant source and feed quality may vary from batch to batch). Thus, it is unlikely that the seasonal changes in relative abundance of different phyla were related to diet. Rabbits are much more tolerant of colder temperatures and low humidity and they are especially prone to heat stress [9, 46]. In general, Canadian rabbit barns do not have environmental controls for temperature or relative humidity and rabbits are exposed to hot and humid ambient conditions in July and August. The relative reduction in levels of the two beneficial phyla (Firmicutes and Verrucomicrobia), and the relative increase in less beneficial phyla (Proteobacteria) could relate to seasonal-related climate changes and directly impact rabbit health, susceptibility to enteritis, and possibly feed conversion efficiency. Additional studies are needed to explore these possibilities. Studies of potential seasonal differences in gut microbiota of rabbits from other sources are also needed.

A potential limitation of this study is whether the fecal microbiota is reflective of the gastrointestinal microbiota of rabbits in general. Michelland et al. [47] demonstrated no significant differences in bacterial community diversity between soft and hard feces or cecal content in rabbits. In addition, Schoster et al. [48] demonstrated that while significant differences existed between the microbiota of the stomach, duodenum, ileum, large colon, and feces in healthy horses, another hindgut-fermenting species, the microbiota profile remained stable from the cecum to the feces. These studies suggest that, at least in the case of hindgut-fermenting species, the fecal microbiota is representative of the distal gastrointestinal tract. For rabbits, in which the cecal microbiota is of primary consideration for overall animal health, the fecal microbiota provides an accurate and non-invasive method for studying their gastrointestinal health.

This study exclusively characterized the bacterial fecal microbiota of rabbits. It is well known that other prokaryotic and eukaryotic microorganisms, such as viruses, bacteriophages, protozoa, archaea and fungi, are essential components of the gastrointestinal microbiota, and can play significant roles in human and animal health [23, 49]. As pathogenic viruses, in addition to pathogenic bacteria, are significant contributors to rabbit dysbiosis and REC, a greater understanding of the rabbit gut virome would provide further insight into understanding rabbit health.

Lastly, it is unknown whether the organisms identified using next generation sequencing methods are functionally or metabolically active. Future computational studies are needed to specifically examine the metabolomics of the bacteria identified.

## Conclusions

This study characterized the composition of the domestic rabbit fecal microbiota, as well as potential factors influencing its composition, such as rabbit source, age, and season, and the routine use of antimicrobials. By gaining an increased understanding of the rabbit enteric microbiota and factors that influence its composition, rabbit health and welfare can be better managed.

## Methods

### Animals and study approval

Commercial meat rabbit producers were contacted through Ontario Rabbit, a rabbit producer group, and asked to voluntarily participate in the study. Feces from domestic rabbits from 27 commercial farms (representing approximately 25% of large rabbit farms in Ontario, Canada) were collected for this study. Rabbit feces were sampled based on age (does and growers, aka fryers) and fecal samples were collected during summer and winter months. Feces from an additional 62 clinically healthy adult companion rabbits of varying ages and breeds were included. Rabbit fecal samples from one commercial laboratory rabbit vendor and seven laboratory research facilities in Ontario were also included, as were rabbit feces from four shelters in southwestern Ontario. All known history for samples collected is listed in Table 3. In all cases, participation in the study was voluntary, with companion, laboratory, and shelter organizations contacted directly by the researchers of the study.

**Table 3:** History of samples collected by source

Facility number	Sample number	Age group	Sample submission date	Sample type
Commercial				
1	1	Does	Summer 2012	Pooled
	2	Growers	Summer 2012	Pooled
	3	Does	Winter 2013	Pooled
	4	Growers	Winter 2013	Pooled
2	1	Does	Summer 2012	Pooled
	2	Growers	Summer 2012	Pooled
	3	Does	Winter 2014	Pooled
	4	Growers	Winter 2014	Pooled
3	1	Does	Summer 2012	Pooled
	2	Growers	Summer 2012	Pooled
	3	Does	Winter 2013	Pooled
	4	Growers	Winter 2013	Pooled
	5	Does	Winter 2013	Pooled
4	1	Does	Summer 2012	Pooled
	2	Growers	Summer 2012	Pooled
	3	Does	Winter 2013	Pooled
	4	Growers	Winter 2013	Pooled
5	1	Does	Summer 2012	Pooled
	2	Growers	Summer 2012	Pooled
	3	Does	Winter 2013	Pooled
	4	Growers	Winter 2013	Pooled
6	1	Does	Summer 2012	Pooled
	2	Growers	Summer 2012	Pooled
	3	Does	Winter 2014	Pooled
	4	Growers	Winter 2014	Pooled
7	1	Does	Summer 2012	Pooled
	2	Growers	Summer 2012	Pooled
	3	Does	Winter 2014	Pooled
	4	Growers	Winter 2014	Pooled
8	1	Growers	Summer 2013	Pooled
	2	Growers	Summer 2013	Pooled
9	1	Does	Winter 2014	Pooled
	2	Growers	Winter 2014	Pooled
10	1	Does	Summer 2013	Pooled
	2	Growers	Summer 2013	Pooled
	3	Does	Winter 2014	Pooled
	4	Growers	Winter 2014	Pooled
11	1	Does	Winter 2014	Pooled
	2	Growers	Winter 2014	Pooled
	3	Does	Summer 2014	Pooled
	4	Growers	Summer 2014	Pooled
	5	Diarrheic animal	Summer 2014	Individual
12	1	Does	Summer 2013	Pooled
	2	Growers	Summer 2013	Pooled
	3	Does	Winter 2014	Pooled
	4	Growers	Winter 2014	Pooled
13	1	Does	Summer 2013	Pooled
	2	Growers	Summer 2013	Pooled
	3	Does	Winter 2014	Pooled
	4	Growers	Winter 2014	Pooled
14	1	Does	Summer 2013	Pooled
	2	Growers	Summer 2013	Pooled
	3	Does	Winter 2014	Pooled
	4	Growers	Winter 2014	Pooled
15	1	Does	Summer 2013	Pooled
	2	Growers	Summer 2013	Pooled
	3	Does	Winter 2014	Pooled
	4	Growers	Winter 2014	Pooled
16	1	Does	Winter 2014	Pooled
	2	Growers	Winter 2014	Pooled
17	1	Does	Summer 2013	Pooled
	2	Growers	Summer 2013	Pooled
	3	Does	Summer 2014	Pooled
	4	Growers	Summer 2014	Pooled
18	1	Does	Summer 2013	Pooled
	2	Growers	Summer 2013	Pooled
	3	Does	Winter 2014	Pooled
	4	Growers	Winter 2014	Pooled
19	1	Does	Summer 2013	Pooled
	2	Growers	Summer 2013	Pooled
	3	Does	Winter 2014	Pooled
	4	Growers	Winter 2014	Pooled
20	1	Does	Summer 2013	Pooled
	2	Growers	Summer 2013	Pooled
	3	Does	Winter 2014	Pooled
	4	Growers	Winter 2014	Pooled
21	1	Does	Summer 2013	Pooled
	2	Growers	Summer 2013	Pooled
	3	Does	Winter 2014	Pooled
	4	Growers	Winter 2014	Pooled
22	1	Does	Summer 2013	Pooled
	2	Growers	Summer 2013	Pooled
	3	Does	Winter 2014	Pooled
	4	Growers	Winter 2014	Pooled
23	1	Does	Summer 2013	Pooled
	2	Growers	Summer 2013	Pooled
	3	Does	Winter 2014	Pooled
	4	Growers	Winter 2014	Pooled
24	1	Does	Summer 2013	Pooled
	2	Growers	Summer 2013	Pooled
	3	Does	Winter 2014	Pooled
	4	Growers	Winter 2014	Pooled
25	1	Does	Summer 2013	Pooled
	2	Growers	Summer 2013	Pooled
26	1	Does	Winter 2014	Pooled
	2	Growers	Winter 2014	Pooled
	3	Does	Summer 2014	Pooled
	4	Growers	Summer 2014	Pooled
27	1	Does	Winter 2014	Pooled
	2	Growers	Winter 2014	Pooled
Laboratory				
1	1 (2014)	Adults	Winter 2014	Pooled
	2 (2015)	Adults	Spring 2015	Pooled
2	1	Adults	Winter 2014	Pooled
3	1	Fryers	Winter 2014	Pooled
	2	Adults	Winter 2014	Pooled
	3	Adults	Winter 2014	Pooled
4	1	Adults	Spring 2015	Pooled
5	1	Adults	Spring 2015	Pooled
	2	Adults	Spring 2015	Pooled
6	1 (Site 1)	Adults	Spring 2015 (Site 1)	Pooled
	2 (Site 2)	Adults	Spring 2015 (Site 2)	Pooled
7	1 (New arrivals)	Adults	Spring 2015	Pooled
	2 (8 weeks following arrival)	Adults	Spring 2015	Pooled
8	1	Adults	Spring 2015	Pooled
Shelter				
1	1	Adult	Fall 2013	Individual
	2	Adult	Fall 2013	Individual
	3	Adult	Fall 2013	Individual
2	1	Adult	Spring 2015	Individual
3	1	Adult	Spring 2015	Individual
	2	Adult	Spring 2015	Individual
	3	Adult	Spring 2015	Individual
	4	Adult	Spring 2015	Individual
	5	Adult	Spring 2015	Individual
4	1	Adult	Spring 2015	Individual
	2	Adult	Spring 2015	Individual
	3	Adult	Spring 2015	Individual
	4	Adult	Spring 2015	Individual
	5	Adult	Spring 2015	Individual
	6	Adult	Spring 2015	Individual
Companion				
1				Individual
2				Individual
3				Individual
4				Individual
5				Individual
6				Individual
7				Individual
8				Individual
9				Individual
10				Individual
11				Individual
12				Individual
13				Individual
14				Individual
15				Individual
16				Individual
17				Individual
18				Individual
19				Individual
20				Individual
21				Individual
22				Individual
23				Individual
24				Individual
25				Individual
26				Individual
27				Individual
28				Individual
29				Individual
30				Individual
31				Individual
32				Individual
33				Individual
34				Individual
35				Individual
36				Individual
37				Individual
38				Individual
39				Individual
40				Individual
41				Individual
42				Individual
43				Individual
44				Individual
45				Individual
46				Individual
47				Individual
48				Individual
49				Individual
50				Individual
51				Individual
52				Individual
53				Individual
54				Individual
55				Individual
56	1	Adult	Spring 2015	Individual
	2	Adult	Spring 2015	Individual
57	1	Adult	Spring 2015	Individual
	2	Adult	Spring 2015	Individual
	3	Adult	Spring 2015	Individual
58	1	Adult	Spring 2015	Individual
59	1	Adult	Spring 2015	Individual

### Fecal sample collection

Hard feces of commercial meat rabbits (*n* = 100 samples) and laboratory rabbits (*n* = 14 samples) were collected from pans beneath rabbit home cages. In all cases, attempts were made to collect the freshest samples from the top layer of feces in the collecting pan (i.e., the most recently voided samples) or by placing a clean pan within 48 h prior to collection. Pooled fecal samples were collected from 3 separate cages per age group that were well-dispersed throughout each barn or facility and mixed thoroughly in sterile plastic bags. For commercial rabbits, one sample bag was submitted for each of two age groups, growers (aged 5–12 weeks) and does (reproductively active, adult, females) per farm. Samples from commercial rabbits were collected during summer (July-Aug) and winter (Feb-Mar) months, when possible. Producers were also asked to verbally report any antimicrobial use, both routine and sporadic, at the time of collection; this information was later confirmed by email or telephone follow-up. At research facilities, when possible, multiple samples (up to 3) were collected from the same facility if there were enough number of rabbits kept in the same room to allow for multiple collections (1 facility), rabbits were kept in multiple rooms or multiple sites throughout the facility (1 facility), significantly different age groups were present within the facility (1 facility), an opportunity was provided to collect samples in repeated years (1 facility), and an opportunity occurred to collect samples immediately following rabbit shipping/arrival to the facility and then following a period of acclimation (1 facility). Fecal samples from companion rabbits were collected from clinically normal rabbits whose owners attended a regional rabbit exposition (*n* = 55) and from healthy patients visiting the Ontario Veterinary College’s Avian and Exotic Service for dental procedures (*n* = 7). Samples were chilled or placed on ice and transported for coding and processing to a central laboratory where 0.2 g of feces from each sample were isolated for DNA extraction while the remaining sample was frozen at -80 °C.

### DNA extraction and quality control

DNA extraction was performed using the E.N.Z.A. Stool DNA Kit (Omega Bio-Tek Inc., Doraville, Georgia, USA) following the manufacturer’s protocol for pathogen detection. Quantity and quality of extracted nucleic acids were assessed by spectrophotometry (NanoDrop, Roche, Mississauga, ON, Canada).

### 16S rRNA gene amplification, purification, and sequencing

Using the protocol described by Caporaso et al. [50] and the primers recommended by Klindworth et al. [51], the V4 region of the 16S rRNA gene was amplified by PCR using the forward primer S-D-Bact-0564-a-S-15 (5’-AYTGGGYDTAAGNG-3′) and the reverse primer S-D-Bact-0785-b-A-18 (5’-TACNVGGGTATCTAATCC-3′), with an expected product size of 240 bp. These forward and reverse primers contained a region that overlapped the Illumina forward and reverse sequencing primers (TCGTCGGCAGCGTCAGATGTGTATAAGAGACAG and GTCTCGTGGGCTCGGAGATGTGTATAAGAGACAG, respectively), allowing them to anneal to the primers containing the Illumina adaptors plus the 8 bp identifier indices (forward: AATGATACGGCGACCACCGAGATCTACAC-index-TCGTCGGCAGCGTC; reverse:CAGCAGAAGACGGCATACGAGAT-index-GTCTCGTGGGCTCGG). For each extracted sample, a 25uL reaction was performed using 12.5uL KAPA Ready Mix, 9.0uL sterile water, 0.5uL each forward and reverse primers (10pM/uL), and 2.5uL DNA template (5 ng/uL). The PCR conditions were as follows: 1) 3 min at 94 °C for denaturation, 2) 45 s at 94 °C for denaturation, 3) 60 s at 53 °C for denaturation, 4) 1.5 min at 72 °C for elongation, and 5) 10 min at 72 °C. Steps 2–4 were repeated for a total of 27 cycles. PCR products were stored at 4 °C until purification was completed. Electrophoresis of PCR products was conducted in 2% agarose gel to evaluate the products for the presence of bands of the appropriate length (~ 240 bp). Purification of PCR products was conducted using Agencourt AMPure XP (Beckman Coulter Inc., Mississauga, ON, Canada); 20uL of AMPure XP was mixed with the PCR product on a 96 well plate. Following 2 min at room temperature, the mixture was transferred to a magnetic plate, left for 2 min, and then the supernatant was discarded. Beads were then washed with 200uL freshly made 80% ethanol twice, air dried for 10 min, and separated from the magnet. The beads were then rinsed with 52.5uL of 10 mM Tris pH 8.5 buffer, placed back onto the magnetic plate, and 50uL of the supernatant of each sample was transferred to a new tube.

A second reaction was performed using 2.5uL purified product, 12.5uL KAPA Ready mix, 9.0uL sterile water, and 1.0uL each Illumina Forward Index Primer (I501-I508 or S513, S15-S18, S20-S22) (10pM/uL) and Illumina Reverse Index Primer (I701–712 or N716, N718-N729) for each sample. An additional PCR was conducted with the following conditions: 1) 3 min at 94 °C, 2) 45 s at 94 °C, 3) 60 s at 50 °C, 4) 1.5 min at 72 °C, and 5) 10 min at 72 °C. Steps 2–4 were repeated for a total of 8 cycles completed. Again, the second PCR product was purified using AMPure XP using a similar procedure to that described above, with 40uL of AMPure XP and 37uL 10 mM Tris pH 8.5 Buffer. An evaluation of final products was completed using electrophoresis in 2% agarose gel, again observing for bands of the appropriate length.

When bands were not visible or of an inappropriate length, spectrophotometry was performed to identify nucleic acid quantity; adjustments to the volumes of KAPA Ready mix, DNA product, and sterile water were made based on the quantity of nucleic acids identified as needed to produce bands of the appropriate size.

Purified samples were normalized to a final concentration of 12.5 nM and submitted for sequencing to the University of Guelph’s Advanced Analysis Centre. An Illumina MiSeq (San Diego, California, USA) and 2 × 250 chemistry were utilized for sequencing of the library pool.

### Microbiota assessment and analyses

Analysis of the sequencing results was conducted using Mothur software (v1.35) [52]. Paired-end reads were aligned and sequences greater than 244 bp or less than 237 bp in length were removed from the data set. Any sequences containing long runs (> 8 bp) of holopolymers, as well as ambiguous base calls, sequences that did not align with the 16S rRNA gene V4 region, or that were identified as chimeras were also removed. Sequences from mitochondria, chloroplasts, Archaea, and eukaryotes were also removed. Sequences were binned into operational taxonomic units (OTUs) at the 3% dissimilarity level using nearest neighbour clustering using open OTU picking. Comparison of relative abundances of different taxa was conducted using one-way ANOVAs within the JMP 12 Response Screening Platform (SAS Institute Inc., Cary, NC, USA). Adjustment of *p* values to account for false discovery rate (FDR) was performed using the Benjamini-Hochberg technique. Analysis was performed both with facility (i.e., where the animal came from) named as a random effect and without. Follow-up post-hoc testing of taxa identified as significantly different between groups was conducted using the Steel-Dwass nonparametric test. Results were considered significant when adjusted *P* values were ≤ 0.05. Differences in microbiota composition based on age (fryers versus does) and season (summer versus winter), were calculated exclusively for commercial meat rabbits.

For subsequent analysis, subsampling was performed to normalize sequence numbers. Good’s Coverage was used to assess coverage to ensure that the microbial population was adequately represented within the samples. Population richness was assessed using Chao1 to identify the number of different organisms present within each sample and the inverse Simpson’s index was used to describe population diversity to determine the abundance of the different organisms present within each sample. The Jaccard index (which measures community membership by comparing the number of shared OTUs, but not their abundance) and Yue and Clayton measure of dissimilarity (which measures community structure by examining shared OTUs and their relative abundances) were used to assess population dissimilarity and to create dendrograms to compare how closely related the microbial communities of each sample were to one another. These were compared between groups for source, age and season using unweighted UniFrac (which measures the phylogenetic distance between communities based on membership), as well as parsimony tests (which examines community membership similarily). Figures were created using FigTree (v1.4.2) (http://tree.bio.ed.ac.uk) and JMP 12. Principal coordinate analysis (PCoA) was also performed to visualize data similarities and dissimilarities between microbial communities and figures were created in JMP 12. LEfse was also performed to identify OTUs that were differentially abundant between groups.

## Additional files

Additional file 1: **Table S1.** Relative abundance of predominant (≥ 1%) taxonomic classifications of bacteria isolated from the feces of domestic rabbits (*n* = 168). (DOCX 14 kb)

Additional file 2: **Table S2.** Relative abundance and FDR *p*-values for significantly different bacterial phyla, classes, orders, families, and genera in the rabbit fecal microbiota when compared between commercial (*n* = 86), companion (*n* = 54), laboratory (*n* = 14), and shelter (*n* = 14) rabbit samples. (DOCX 24 kb)

## Abbreviations

ANOVA: Analysis of variance
FDR: False discovery rate
GI: Gastrointestinal
LDA: Linear discriminant analysis
LEfSe: Linear discriminant analysis effect size
OTU: Operational taxonomic units
PCoA: Principal coordinate analysis
REC: Rabbit enteritis complex
