# Supplementary material for: Comparison of the fecal microbiota of domestic commercial meat, laboratory, companion, and shelter rabbits (Oryctolagus cuniculi)
Source: BMC Vet Res. 2018 Apr 27;14:143. doi: 10.1186/s12917-018-1464-6 (PMC5924505; doi:10.1186/s12917-018-1464-6)
Supplement: Supplementary file 1 — Table S1. Relative abundance of predominant (≥ 1%) taxonomic classifications of bacteria isolated from the feces of domestic rabbits (n = 168). (DOCX 14 kb) [file 12917_2018_1464_MOESM1_ESM.docx]

Additional Table 1. Relative abundance of predominant (>1%) taxonomic classifications of bacteria isolated from the feces of domestic rabbits (n=168).

a) Phylum

| Phylum | Relative Abundance |
| --- | --- |
| Firmicutes | 66.42% |
| Verrucomicrobia | 14.05% |
| Proteobacteria | 9.54% |
| Unclassified at phylum level | 6.87% |
| Bacteroidetes | 1.54% |

b) Class

| Class | Relative Abundance |
| --- | --- |
| Clostridia | 55.25% |
| Verrucomicrobia | 14.85% |
| Unclassified Firmicute | 7.90% |
| Unclassified at phylum | 7.77% |
| Gammaproteobacteria | 4.22% |
| Bacilli | 2.68% |
| Betaproteobacteria | 1.13% |
| Bacteroidia | 1.03% |

c) Order

| Order | Relative Abundance |
| --- | --- |
| Clostridiales | 54.79% |
| Verrucomicrobiales | 14.85% |
| Unclassified Firmicute | 7.90% |
| Unclassified at phylum | 7.77% |
| Pseudomonadales | 1.89% |
| Bacillales | 1.49% |
| Lactobacillales | 1.19% |
| Xanthomonadales | 1.17% |
| Bacteroidales | 1.03% |

d) Family

| Family | Relative Abundance |
| --- | --- |
| Unclassified Halanaerobiales (Firmicutes) | 20.77% |
| Ruminococcaceae | 20.03% |
| Verrucomicrobiaceae | 13.92% |
| Lachnospiraceae | 12.83% |
| Unclassified Firmicutes | 7.29% |
| Unclassified Phylum | 6.87% |
| Xanthomonadaceae | 1.84% |
| Pseudomonadaceae | 1.52% |
| Alcaligenaceae | 1.08% |

e) Genus

| Genus | Relative Abundance |
| --- | --- |
| Unclassified Clostridiales (Firmicutes) | 20.77% |
| Unclassified Ruminococcaceae (Firmicutes) | 10.86% |
| *Persicirhabdus* | 9.32% |
| Unclassified Lachnospiracea (Firmicutes) | 8.26% |
| Unclassified Firmicutes | 7.29% |
| Unclassified Phylum | 6.87% |
| Ruminococcus | 4.93% |
| Unclassified Verrucomicrobiaceae (Verrucomicrobia) | 2.66% |
| *Akkermansia* | 1.94% |
| *Ignatzschineria* | 1.46% |
| *Clostridium_XlVa* | 1.23% |
