# Supplementary material for: Comparison of the fecal microbiota of domestic commercial meat, laboratory, companion, and shelter rabbits (Oryctolagus cuniculi)
Source: BMC Vet Res. 2018 Apr 27;14:143. doi: 10.1186/s12917-018-1464-6 (PMC5924505; doi:10.1186/s12917-018-1464-6)
Supplement: Supplementary file 2 — Table S2. Relative abundance and FDR p-values for significantly different bacterial phyla, classes, orders, families, and genera in the rabbit fecal microbiota when compared between commercial (n = 86), companion (n = 54), laboratory (n = 14), and shelter (n = 14) rabbit samples. (DOCX 24 kb) [file 12917_2018_1464_MOESM2_ESM.docx]

Additional Table 2. Relative abundance and FDR p-values for significantly different bacterial phyla, classes, orders, families, and genera in the rabbit fecal microbiota when compared between commercial (n=86), companion (n=54), laboratory (n=14), and shelter (n=14) rabbit samples.

a) Phylum

|  |  | Relative Abundance - Commercial | Relative Abundance - Companion | Relative Abundance - Laboratory | Relative Abundance - Shelter | FDR p-value |
| --- | --- | --- | --- | --- | --- | --- |
| Lentisphaerae |  | <0.01%^a,c^ | 0.04%^d,b^ | <0.01%^a^ | 0.01%^d^ | 0.01 |
| Proteobacteria |  | 14.04%^a,b^ | 4.0%^d^ | 4.19%^d^ | 7.52% | <0.01 |
| Verrucomicrobia |  | 8.89%^a,b,c^ | 22.51%^b,c,d^ | 14.57%^a,d^ | 13.49%^a,d^ | <0.01 |

b) Class

|  |  | Relative Abundance - Commercial | Relative Abundance - Companion | Relative Abundance - Laboratory | Relative Abundance - Shelter | FDR p-value |
| --- | --- | --- | --- | --- | --- | --- |
| Unclassified Acidobacteria* | | <0.01%^a,b,c^ | <0.01%^d^ | <0.01%^d^ | 0.01%^d^ | 0.05 |
| Lentisphaeria |  | <0.01%^a,c^ | 0.04%^b,d^ | <0.01%^a^ | 0.01%^d^ | 0.01 |
| Betaproteobacteria |  | 2.43%^a,b,c^ | 0.47%^d^ | 0.63%^d^ | 0.39%^d^ | <0.01 |
| Deltaproteobacteria |  | 0.24%^a,c^ | 0.44%^b,d^ | 0.18%^a,c^ | 0.49%^b,d^ | <0.01 |
| Epsilonproteobacteria |  | 0.16%^a,b^ | 0.38%^b,d^ | 0.07%^a,d^ | 0.21% | 0.02 |
| Gammaproteobacteria |  | 9.44%^a,b,c^ | 1.50%^d^ | 1.27%^d^ | 3.74%^d^ | <0.01 |
| Verrucomicrobiae |  | 8.74%^a,b,c^ | 22.39%^c,d^ | 14.51%^c,d^ | 13.38%^a,b,d^ | <0.01 |
| Bacilli |  | 3.79%^a,b^ | 1.27%^d^ | 1.11%^d^ | 4.96% | 0.03 |
| Erysipelotrichia |  | 0.62%^a^ | 0.16%^d^ | 0.23% | 0.38% | <0.01 |

c) Order

|  |  | Relative Abundance - Commercial | Relative Abundance - Companion | Relative Abundance - Laboratory | Relative Abundance - Shelter | FDR p-value |
| --- | --- | --- | --- | --- | --- | --- |
| Unclassified Acidobacteria* | | 0.00%^a,b,c^ | <0.01%^d^ | <0.01%^d^ | 0.01%^d^ | 0.05 |
| Thermales |  | <0.01%^a^ | 0.01%^d^ | 0.01% | 0.01% | 0.01 |
| Victivallales |  | <0.01^a,c^ | 0.04%^b,d^ | <0.01%^a^ | 0.01%^d^ | 0.01 |
| Sneathiellales |  | 0.00%^c^ | <0.01% | <0.01% | <0.01%^d^ | <0.01 |
| Unclassified Alphaproteobacteria* (Proteobacteria) | | 0.53%^a,b^ | 0.26%^b,c,d^ | 1.06%^a,d^ | 1.12%^a^ | <0.01 |
| Burkholderiales |  | 2.17%^a,b,c^ | 0.12%^d^ | 0.05%^d^ | 0.05%^d^ | <0.01 |
| Unclassified Betaproteobacteria* (Proteobacteria) | | 0.24%^b^ | 0.33%^b^ | 0.57%^a,d^ | 0.34% | <0.01 |
| Desulfovibrionales |  | 0.15%^a,b^ | 0.29%^b,d^ | 0.08%^a,d^ | 0.21% | <0.01 |
| Unclassified Deltaproteobacteria* (Proteobacteria) | | 0.03% | 0.05% | 0.03% | 0.12% | 0.01 |
| Campylobacterales |  | 0.16%^a,b^ | 0.38%^b,d^ | 0.07%^a,d^ | 0.21% | 0.02 |
| Aeromonadales |  | 0.18%^a,b,c^ | 0.01%^d^ | <0.01%^d^ | 0.01%^d^ | 0.03 |
| Pseudomondales |  | 3.80%^a,b^ | 0.78%^d^ | 0.60%^d^ | 2.69% | <0.01 |
| Xanthomonadales |  | 3.51%^a,b,c^ | 0.03%^d^ | 0.02%^d^ | 0.02%^d^ | <0.01 |
| Unclassified Opitutae* (Verrucomicrobia) | | <0.01%^a^ | 0.01%^d^ | <0.01% | 0.01% | 0.02 |
| Verrucomicrobiales |  | 8.74%^a,b,c^ | 22.39%^b,c,d^ | 14.52%^a,d^ | 13.38%^a,d^ | <0.01 |
| Lactobacillales |  | 2.56%^a,b,c^ | 0.22%^d^ | 0.23%^d^ | 1.14%^d^ | <0.01 |
| Erysipelotrichales |  | 0.62%^a^ | 0.16%^d^ | 0.23% | 0.38% | <0.01 |

d) 20 most prevalent families:

|  |  | Relative Abundance - Commercial | Relative Abundance - Companion | Relative Abundance - Laboratory | Relative Abundance - Shelter | FDR p-value |
| --- | --- | --- | --- | --- | --- | --- |
| Lachnospiraceae |  | 12.20%^c^ | 14.85%^c^ | 13.30%^c^ | 8.51%^a,b,d^ | 0.01 |
| Verrucomicrobiaceae |  | 8.74%^a,b,c^ | 22.39%^b,c,d^ | 14.52%^a,d^ | 13.38%^a,d^ | <0.01 |
| Xanthomonadaceae |  | 3.51%^a,b,c^ | 0.02%^d^ | 0.01%^d^ | 0.02%^d^ | <0.01 |
| Pseudomonadaceae |  | 2.89%^a,b,c^ | 0.22%^d^ | 0.46%^d^ | 2.15%^d^ | 0.03 |
| Alcaligenaceae |  | 2.04%^a,b,c^ | 0.07%^d^ | 0.01%^d^ | 0.01%^d^ | <0.01 |
| Carnobacteriaceae |  | 1.41%^a,b,c,^ | 0.05%^d^ | 0.03%^d^ | 0.15%^d^ | 0.01 |
| Aerococcaceae |  | 0.81%^a,b,c^ | 0.09%^d^ | 0.10%^d^ | 0.88%^d^ | 0.01 |
| Erysipelotrichaceae |  | 0.62%^a^ | 0.16%^d^ | 0.23% | 0.38% | <0.01 |
| Unclassified Alphaproteobacteria* (Proteobacteria) | | 0.53%^a,b^ | 0.26%^b,c,d^ | 1.06%^a,d^ | 1.12%^a^ | <0.01 |
| Unclassified Nitrosomonadales* (Proteobacteria) | | 0.24%^b^ | 0.33%^b^ | 0.57%^a,d^ | 0.34% | <0.01 |
| Rikenellaceae |  | 0.20%^b^ | 0.16%^b^ | 0.34%^a,d^ | 0.67% | 0.03 |
| Aeromonadaceae |  | 0.18%^a,b,c^ | <0.01%^d^ | <0.01%^d^ | <0.01%^d^ | 0.03 |
| Campylobacteraceae |  | 0.15%^a,b^ | 0.38%^b,d^ | 0.07%^a,d^ | 0.19% | 0.03 |
| Unclassified Lactobacillales* (Firmicutes) | | 0.13%^a,b,c^ | <0.01%^d^ | <0.01%^d^ | <0.01%^d^ | <0.01 |
| Oceanospirillaceae |  | 0.12%^a,b,c^ | <0.01%^d^ | <0.01%^d^ | 0.01%^d^ | 0.03 |
| Desulfovibrionaceae |  | 0.10%^a,b^ | 0.21%^b,d^ | 0.05%^a,c,d^ | 0.14%^b^ | <0.01 |
| Unclassified Desulfovibrionales* (Proteobacteria) | | 0.04%^a,b^ | 0.08%^b,d^ | 0.02%^a,c,d^ | 0.07%^b^ | 0.04 |
| Clostridiales_Incertae  _Sedis_XIII | | 0.04% | 0.03% | 0.08%^c^ | 0.06%^b^ | 0.02 |
| Microbacteriaceae |  | 0.04%^a,b^ | <0.01%^d^ | 0.00%^c,d^ | <0.01%^b^ | <0.01 |
| Unclassified Deltaproteobacteria* (Proteobacteria) | | 0.03% | 0.05% | 0.03% | 0.12% | <0.01 |

e) 20 most prevalent genera:

|  |  | Relative Abundance - Commercial | Relative Abundance - Companion | Relative Abundance - Laboratory | Relative Abundance - Shelter | FDR p-value |
| --- | --- | --- | --- | --- | --- | --- |
| Unclassified Lachnospiaceae* (Firmicutes) | | 7.74%^c^ | 9.65%^c^ | 9.09%^c^ | 5.36%^a,b,d^ | 0.01 |
| *Persicirhabdus* |  | 5.94%^a,b^ | 14.55%^d^ | 10.40%^d^ | 9.41% | <0.01 |
| *Ignatzschineria* |  | 2.80%^a,b,c^ | <0.01%^d^ | 0.01%^d^ | 0.01%^d^ | 0.03 |
| Unclassified Verrucomicrobiaceae* (Verrucomicrobia) | | 1.79%^a,b^ | 4.57%^b,d^ | 0.94%^a,c,d^ | 2.43%^b^ | <0.01 |
| *Atopostipes* |  | 1.32%^a,b,c^ | 0.04%^d^ | <0.01%^d^ | 0.13%^d^ | 0.01 |
| *Paenalcaligenes* |  | 1.23%^a,b,c^ | 0.05%^d^ | <0.01%^d^ | <0.01^d^ | <0.01 |
| *Blautia* |  | 1.17%^a,b,c^ | 0.88%^d^ | 0.73%^d^ | 0.47%^d^ | 0.03 |
| *Akkermansia* |  | 1.00%^a,b^ | 3.27%^d^ | 3.17%^d^ | 1.54% | 0.02 |
| *Clostridium_XlVa* |  | 0.94% | 1.91% | 1.15% | 0.52% | 0.02 |
| Unclassified Alphaproteobacteria* (Proteobacteria) | | 0.53%^a,b^ | 0.26%^b,c,d^ | 1.06%^a,d^ | 1.12%^a^ | <0..01 |
| Unclassified Aerococcaceae* (Firmicutes) | | 0.47%^a,b,c^ | <0.01%^d^ | <0.01%^d^ | 0.01%^d^ | <0.01 |
| *Anaerostipes* |  | 0.32%^a,c^ | 0.10%^b,d^ | 0.47%^a,c^ | 0.15%^b,d^ | 0.01 |
| *Erysipelothrix* |  | 0.26%^a,b,d^ | <0.01%^d^ | <0.01%^d^ | <0.01%^d^ | 0.01 |
| Unclassified Erysipelotrichaceae* (Firmicutes) | | 0.25%^a^ | 0.09%^b,d^ | 0.17%^a^ | 0.31% | 0.02 |
| *Facklamia* |  | 0.25%^a,b,c^ | 0.02%^d^ | 0.03%^d^ | 0.02%^d^ | 0.03 |
| Unclassified Betaproteobacteria* (Proteobacteria) | | 0.24%^b^ | 0.33%^b^ | 0.57%^a,d^ | 0.34% | <0.01 |
| *Alistipes* |  | 0.19%^b^ | 0.15%^b^ | 0.33%^a,d^ | 0.35% | 0.03 |
| *Oceanisphaera* |  | 0.18%^a,b,c^ | <0.01%^d^ | <0.01%^d^ | <0.01%^d^ | 0.04 |
| *Butyricicoccus* |  | 0.15%^a,b,c^ | 0.03%^b,d^ | 0.04%^a,d^ | 0.05%^d^ | 0.02 |
| Unclassified Lactobacillales* (Firmicutes) | | 0.13%^a,b,c^ | <0.01%^d^ | <0.01%^d^ | <0.01%^d^ | <0.01 |

*For samples that could not be classified at the specified phylogenetic level, the highest level has been specified with the associated phylum in brackets if classification was possible beyond the phylum level

^a^Significantly different (p<0.05) from companion rabbits as identified by the Steel-Dwass test

^b^Significantly different (p<0.05) from laboratory rabbits as identified by the Steel-Dwass test

^c^Significantly different (p<0.05) from shelter rabbits as identified by the Steel-Dwass test

^d^Significantly different (p<0.05) from commercial meat rabbits as identified by the Steel-Dwass test
